# Salutary factors and hospital work environments: a qualitative descriptive study of nurses in Sweden

**DOI:** 10.1186/s12912-020-00521-y

**Published:** 2020-12-20

**Authors:** Nunstedt Håkan, Eriksson Monica, Obeid Ayman, Hillström Lisbeth, Truong Anh, Pennbrant Sandra

**Affiliations:** Department of Health Sciences, University West, Trollhättan, Sweden

**Keywords:** Salutogenesis, Sense of coherence, Comprehensibility, Manageability, Meaningfulness, Nurses, Sustainable working life

## Abstract

**Background:**

Extensive research describes how nurses experience their work environment. The conditions are described as stressful and dissatisfying with nurses intending to leave their workplace. Knowledge about the personal perception regarding why nurses consider leaving the hospital workplace is limited. The purpose of this study was to understand why hospital nurses remain in their workplace, which facilitates their continuation in the profession.

**Objective:**

The objective was to explore and describe factors explaining why hospital nurses remain in the workplace.

**Methods:**

This was a descriptive qualitative study with a purposive sample of hospital nurses in Sweden. The salutogenic theory was the basis for the interview guide and the semi-structured questions. Individual interviews were conducted in a hospital in western Sweden. Content analysis was performed to organize the coded data according to the sense of coherence.

**Results:**

Data saturation was achieved with 12 interviews. Within the three themes of coherence (comprehensibility, manageability, and meaningfulness), ten subthemes were categorized from the data as follows: job satisfaction and fun at work, acknowledgement and productivity, togetherness and team security, manageable workload, variable work and challenging situations, workplace and personal space balance, collaboration and supportive leadership, valued role and good work, commitment and involvement, and pride in the professional role.

**Conclusions:**

The main findings of this study have shown the critical importance of being in a meaningful, comprehensible and manageable work context that supports nurses in maintaining their professional identity.

## Background

Turnover among nurses is a global concern that negatively affects health services. Hospital settings are characterized by high complexity owing to the interaction between patients, nurses and the organization. Nurses play a pivotal role in delivering care to hospitalized patients. In the European context, there is a serious nursing shortage in most countries [1]. The “Registered nurse forecasting in Europe study” [2] brought together researchers from 12 European countries to generate a large evidence basis about nursing workforce issues [3, 4]. As many as 20–50% of nurses in each country intended to leave their job during the next year. In general, 9% of nurses intended to leave nursing, most of them after only a few years in the profession [3, 4]. In Canada, work environment variables explained 45.5% of the variance in nurses’ intent-to-leave scores [3]. Further contributing to the problem is that the ageing segment of the population is rapidly expanding and consuming more health services with fewer new nurses entering the workforce. As such, providing a healthy work environment to retain nurses in their workplace is essential for sustaining the profession. The present study seeks to understand the factors that can increase both the professional longevity of nurses working in hospitals and nurses’ willingness to remain in work and the profession.

### Previous research

There is extensive research on how nurses experience their working conditions. Nurses work in environments that contribute to high stress, job dissatisfaction, burnout [5], intention to leave the workplace [6, 7], and intention to leave the profession [8]. As a result, nurses frequently intend to leave their workplace and sometimes the profession. In a cross-sectional study in a Swiss hospital setting, one in six nurses thought frequently about leaving the profession. According to Hämmig [8], temporal, physical, emotional and mental workloads as well as job stressors were strongly and positively associated with burnout symptoms. A similar scenario was seen among Taiwanese clinical nurses, where five main paths were found from job stress to intention to leave the hospital [9]. In these nurses, job stress directly affected job satisfaction and depressed mood, which in turn affected intention to leave the hospital. The intention to leave the hospital preceded the intention to leave the profession. These results highlight how important it is for nurses to be able to deal with stress in both the short and the long term.

Posttraumatic stress disorder (PTSD) was the focus of a study among nurses working in a variety of sub-specialties and from 12 different countries [10]. Findings from this integrative review revealed that PTSD is a growing concern in the nursing profession. Four themes emerged from the synthesis of factors that influenced PTSD among nurses: workplace matters, relationship matters, the toll of caregiving and interpersonal strengths. These overarching themes captured a multitude of factors that occur across three levels of influence: organizational, interpersonal and intrapersonal. These four themes are connected to ideas from stress management and resource-oriented research, which highlight people’s ability to manage stress and stay well [11]. It is becoming increasingly important to ask not only how nurses survive at work but also how they thrive [12–14].

One way to look at this is to explore how nurses create and maintain a sense of coherence (SOC) and find strategies for managing stress [13]. Salutogenesis is a dynamic, flexible approach continually focused on managing stress [14].

### Theoretical framework

The purpose of this study was to understand why hospital nurses remain in their workplace to better inform the development of sustainable work environments. The salutogenic theory of health guided the present study [11, 13–15]. Salutogenesis is a resource-oriented field of health research that involves understanding people as unique individuals with different resources and competencies. Essential to salutogenic theory is understanding health as a process that exists along a continuum: the health/disease continuum. This is a way of looking at health as a process, as opposed to seeing it as a dichotomy between health and disease. The key concepts are SOC, which is the ability to comprehend the whole situation, the capacity to use available resources, and the generalized and specific resistance resources against stress (GRRs/SRRs) [11, 14]. This capacity is a combination of people’s ability to assess and understand the situation they are in, to find meaning in investing the energy needed to move in a health-promoting direction and to manage the situation – that is, comprehensibility, meaningfulness, and manageability.

In a Swedish context, the level of the three dimensions of SOC was shown to vary. Manageability was shown to be the weakest and to decrease the total SOC, and the meaningfulness dimension was shown to be the strongest [16]. On a national level, nurses reported weaker SOC than the general population, but in an international comparison, they reported stronger SOC, and hospital nurses were reported to find their work difficult to manage but meaningful [14]. Sasso and colleagues [17] described “push and pull” factors involved in nurses’ intention to leave their job. Dissatisfaction was the most important reason for intention to leave the job (35.5%), and among the dissatisfied, 33.1% intended to leave the nursing profession. Push factors included understaffing, emotional exhaustion and poor patient safety. Pull factors for staying included positive perceptions of the quality of care, patient safety and performing core nursing activities.

## Methods

### Study design

The study design was adopted to gain a deeper understanding of why hospital nurses remain in the workplace [15]. The study is qualitative and descriptive in design [18, 19]. Taking this qualitative approach is appropriate for gathering a maximum amount of information within a particular domain [20]. The study was conducted in four phases: 1) a literature review of previous research focusing on salutary factors that have been shown to support sustainability in nurses’ working life; 2) interviews with questions derived from the salutogenic theory and its core concept SOC [15]; seven question areas were identified that formed the basis for developing the interview guide (the guide was specially developed for this study); 3) a qualitative content analysis of the data by an inductive (data-driven) approach [21]; and 4) a deductive approach for theoretically discussing the findings [13].

### Setting and sample

The study was conducted at a hospital in western Sweden. The hospital includes four speciality areas: emergency medicine, specialist medicine, surgical care and adult psychiatric inpatient care. The interview inclusion criteria were understanding and speaking Swedish and at least 5 years of experience working as a nurse. Within the four speciality areas, there are several departments and clinics where physicians, nurses, physiotherapists and occupational therapists work. A purposive sampling method was used. The participants were recruited by sending an informational letter to the unit managers, who in turn invited their staff to participate in the study. The nurses who wished to participate were encouraged to contact the first author (HN) by e-mail. The nurses who consented to participate were contacted by telephone by an interviewer to schedule a time for the interview.

### Data collection

Written informed consent was obtained before starting each interview. The interviewers had previous experience conducting interviews for research purposes. Data collection took place through in-depth face-to-face interviews conducted at a secluded location the respondent was asked to choose at the hospital where the participants worked. The interview guide was employed, using semi-structured and open-ended questions covering factors important to explaining why nurses remain in work and the profession (see examples of items in Table 1). During the interviews, the participants were asked about factors that positively affect job satisfaction, factors that cause them to remain in the profession, organizational and individual conditions that are important to them remaining in working life, their learning experiences and changes they wish to see in their future career.

**Table 1:** Question areas and interview questions

Question areas	Example of questions
1. Job satisfaction	▪ What does job satisfaction mean for you? ▪ What makes you feel good at work? Can you give some examples? ▪ Can you describe what a good day at work looks like?
2. Professional role	▪ How do you see your professional role as a nurse? What does it mean to you? ▪ Are you proud to be a nurse? In what way are you proud to be a nurse? Describe!
3. Job engagement	▪ What motivates you to go to work? Explain! ▪ What drives you to do a good job? Explain! ▪ How do you think the organization is affected by increased engagement among employees? Describe!
4. Belonging in the workplace	▪ What does belonging at work mean for you? Describe! ▪ What do you think an increased belonging at work leads to? ▪ How do you think the organization is affected by an increased belonging in the work among employees?
5. Working conditions and factors for remaining in the profession	▪ What factors have made you want to start working as a nurse? Describe! ▪ What significance does your free time, family and friends have for your work as a nurse? Describe!
6. Opportunities for learning and development in workplace	▪ How do you learn in your work? Describe! ▪ What do you learn in your work? Describe! ▪ Are there other people who influence your learning at work? If yes, describe in what way? ▪ What opportunities are there for you to develop and make a career in your current work situation? Describe!
7. The professional role in the future	▪ How do you see your work and your professional role in the future? In two years, in five years, in ten years? ▪ What new knowledge will nurses need in the future, do you think?

### Data analysis

The collected data were analysed both deductively and inductively using qualitative content analysis [21]. Initially, the entire text was read several times to achieve an overall understanding. In the next step, the texts were reduced into meaning units, that is, parts of the text related to the study aim and research questions. The meaning units were condensed but retained their most significant content. The underlying messages of the condensed meaning units were then labelled with codes. The codes were continually adjusted to make the inductive process more rigorous [21]. Based on the patterns emerging from the analysis, the codes were deductively structured into the dimensions of the SOC – that is, meaningfulness, manageability and comprehensibility – which thus constitute the themes presented in the results section [15]. Subthemes were then identified; they were categorized together into the three main themes and described the latent meaning of the overt statements [21]. An example of how the data were analysed is shown in Table 2. In this way, we also deepened our understanding of the three dimensions of SOC through the analysis of the nurses’ experiences.

**Table 2:** Example of analysis process with sub-themes and themes

Meaning unit	Condensed meaning unit	Code	Subtheme	Theme
That you are enough staff, definitely that is number one, you have to be enough nurses. So enough that both the nurses and the nurses know that they can handle the work and that there is also a buffer if something special should happen. Ideally, there is someone redundant every day, because there is usually someone who is gone, reached a child who is sick, so that you have a small buffer to take off so that you never have to be understaffed for longer periods.	it is to make sure you have enough staff, absolutely. It’s number one	Have a good basic staffing	Manageable workload	Meaningfulness

According to Saunders et al. [22], data saturation differs depending on the type of study and on assumptions concerning whether the data represent an ongoing process. After each interview, we evaluated the data to determine whether there were new patterns or variations in the data to determine saturation.

### Ethical considerations

The nurses were informed about the study both orally and in writing. Informed consent was obtained from the nurses who were willing to participate. They were told that participation was voluntary and that they were free to withdraw from the interview and the study at any time without giving a reason. Permission to conduct the study was obtained from the operational managers.

### Trustworthiness

Trustworthiness can be defined as including credibility, dependability, confirmability and transferability. When evaluating qualitative data, these issues must be considered [20, 21]. To ensure credibility and provide a broad understanding of the problem, participants from several occupational categories with rich experience of caring were included in the study. Credibility [19] was also achieved by choosing individual interviews in which the nurses described what they did to create sustainability in their working life. The procedures for data analysis and generating themes and subthemes have been described above. The analysis process was characterized by critical review on the part of the researchers, who read the interviews and jointly defined the themes and subthemes to ensure the study’s dependability. Confirmability was attained by checking the codes, themes and subthemes against the interviews throughout the analytical process. Confirmability was also strengthened by relating the results to earlier research. The participants’ unique answers and the inductive and deductive process ensure the study’s confirmability. To facilitate transferability, a description of the context, selection and characteristics of the participants has been provided. The methods for identifying and condensing meaning units have also been clearly described, thus allowing readers to easily understand the background of the presented results. Furthermore, excerpts from the interviews have been provided so that the reader can assess whether the findings are transferable to other settings.

The Consolidated Criteria for Reporting Qualitative Research (COREQ) Checklist was used to fulfil the standards of high-quality research.

## Results

Data saturation was achieved after twelve nurses were interviewed. The participants’ sociodemographic variables are shown in Table 3. The interviews lasted between 53 and 106 min (mean 80 min) and were transcribed verbatim; they provided rich data, amounting to more than 200 pages of transcribed text.

**Table 3:** Socio-demographic characteristics of the participants (*N* = 12)

Variable	*N =* 12
**Age** (years)	
Mean	48
Range	39–61
Median	47
**Sex**	
Male	0
Female	12
**Marital status**	
Single	0
Married/cohabitation	12
**Number of years as a nurse, mean**	
Mean	16
Range	5.5–33
Median	13

The results are presented using the three theoretical dimensions of the SOC construct – meaningfulness, manageability and comprehensibility – as themes. The nurses described factors explaining their ability to remain in work and the profession as ten subthemes. (Table 4).

**Table 4:** Salutary factors and work environments explaining why hospital nurses may remain in work. Themes and subthemes

Theme	Subtheme
Meaningfulness	- Job satisfaction and fun at work - Acknowledgement and productivity - Togetherness and team security
Manageability	-Manageable workload -Variable work and challenging situations -Workplace and personal space balance -Collaboration and supportive leadership
Comprehensibility	-Valued role and good work -Commitment and involvement -Pride in professional role

### Meaningfulness

In the workplace, the nurses are in a context that is important to them and feels straightforward. The nurses have a need for acknowledgement from colleagues, patients and their relatives, as such acknowledgement makes the usefulness of their work efforts clear to them and increases the meaningfulness of their work. In this way, the nurses feel they are productive; they can see the results of their own work. The nurses also need to be included in meaningful healthcare teams that are characterized by job satisfaction and humour.

### Job satisfaction and fun at work

The nurses see job satisfaction as one of the most meaningful factors for remaining in the workplace for a long time. This can involve job satisfaction in the team or the joy felt when a patient expresses something positive.

Job satisfaction for me, it's getting someone to smile. Try to spread positive emotions. Be happy and make it easier for others. That gives me job satisfaction; I feel good. A patient who looks at you and says “thank you,” and you can see that everything you did went well (nurse, medical outpatient care).

Having fun at a workplace marked by humour is also something the nurses describe as important to remaining in the workplace for many years. This can entail, for example, using humorous jargon on the team, colleagues having a “twinkle in their eyes” or being able to laugh together. The nurses also describe how important it is to have work that is meaningful; they go to work with a positive feeling.

Now, we have a very fine atmosphere. Our students say that they’ve never been on a ward where they felt so welcome right away, a basic positive feeling and that’s how we want it. We are able to joke and talk to each other. However, I almost see my colleagues more than I see my family (nurse, psychiatric inpatient care).

### Acknowledgement and productivity

According to the nurses, the meaningfulness of their work increases when they are acknowledged. It is often patients who provide this acknowledgement, either directly or when the nurses have contributed to patients’ improved health. However, it can also be colleagues or relatives who acknowledge the nurse.

Every day you get this feedback from patients, whatever it may be, it feels like I've done something good. But, it's not always so, there are others too. Most of the time I’ve done something good every day/…/I feel good about being seen and acknowledged and seeing and acknowledging others (nurse, surgical outpatient care).

The nurses have a great need to feel productive and useful in their work. They can provide self-acknowledgement, especially when patients recover. Then, they see how their involvement has contributed to the positive outcomes.

A workplace where I thrive and feel I go to easily, that I’m useful, needed in any way. That’s what I get from both patients and staff (nurse, psychiatric inpatient care).

### Togetherness and team security

Feeling togetherness and security in the work team is central to nurses’ experience of meaning at work. Cohesion means a great deal to the nurses, that is, working as a team and solving problems together, especially in precarious care situations.

I think togetherness gets a little stronger in crisis situations. I actually think that there are probably many institutions that feel that way, that you get a little tighter during crisis situations. Togetherness becomes stronger in strained situations (nurse, medical inpatient care).

The nurses experience togetherness when they are accepted, involved and part of the work team. They feel they are not alone and have full responsibility for care provision. They feel they are part of a greater whole.

Being accepted by the group, that you’re part of the group, being seen and heard. That people listen to what you have to say and being involved in making decisions. I think it’s important to belong to a group because that's probably what being a nurse means, that you’re independent but still part of the whole. Having colleagues and supporting each other (nurse, medical inpatient care).

### Manageability

High demands are placed on the nurses regarding being able to manage their day-to-day work. These high demands concern both more routine tasks and stressful situations, but the nurses find ways to cope with the different care situations. Factors that make the work situation manageable include receiving support from colleagues and managers, being involved and being able to interact with colleagues and other healthcare professionals. A good balance between work and leisure makes the work more manageable. An additional factor is being able to mentally leave work duties behind at the end of the workday. Having an individual work schedule is valuable for nurses, as it gives them a great deal of flexibility. They also express a need to have a manageable workload, that is, to have control over various care situations. Using these factors, they can build sustainable strategies for managing different care situations and coping with the work, both physically and mentally, for a long time.

### Manageable workload

Nurses typically have a high workload and must prioritize tasks if they are to cope with a variety of care situations. It is important to have enough nurses on the ward to handle the high workload. Having a manageable workload means there is a readiness for unexpected and emergency events.

Enough staff, that is number one, there has to be enough nurses. Enough, so that both nurses and auxiliaries feel they are coping with the work and that there’s a buffer if something happens. That there are extra staff every day, because there’s usually someone who is absent, children who are sick. So that you have a small buffer to rely on, so that you never have to be understaffed for longer periods (nurse, surgical inpatient care).

When the workforce is sufficient and the workload is balanced, there is time for important tasks other than pure physical care or emergency care. This means that nurses have the opportunity to take more time to create care relationships with patients and can perform qualitatively good nursing. This allows them to finish their workday with a higher level of satisfaction.

When we’re sufficiently staffed, just enough workload, then I have time for my responsibilities, I have time to deal with things that are hanging over me. That I get a moment to sit and finish, so I can catch up with my duties (nurse, medical outpatient care).

The workload can vary during the day and across work shifts. If nurses have opportunities to slow their pace during the day, they can take a break between more stressful tasks, which allows a kind of recovery. This recovery, in turn, can provide extra energy to tackle more strenuous situations.

I can work at a fast pace one day because then I know that the next day, maybe, will be a bit calmer, and then I slow down, and I can manage the work. I know that I would have burned out otherwise (nurse, psychiatric inpatient care).

You can work under stress temporarily, you can do it, you can push yourself and then you have to find a recovery phase/…/What the recovery phase looks like is individual, you have to find a recovery phase, a bit every day, and then you can handle more stress again. But I have been stressing for 16 years and then suddenly "Bang!" and I've been there, I know how it feels and I never want to be there again. I’m an expert at learning to say "stop and no," even if I want to (nurse, surgical outpatient care).

### Variable work and challenging situations

Nurses’ professional role involves a variety of everyday tasks and challenges. Although many tasks are based on experience and routine management, nurses are exposed daily to challenges and trials. The nurses feel their tasks are positive challenges in that they create variation, which means working as a nurse never becomes monotonous.

No days are identical, there are encounters with different people, patients and situations. Nothing gets boring, you have to be on the ball all the time, and this makes you more mentally alert (nurse, surgical outpatient care).

I’m very pleased with my situation, that I have a bit of everything; I come in when someone is sick or when there is a shortage of nurses, so it feels good (nurse, psychiatric inpatient care).

The varying tasks mean that the nurses are constantly learning, especially when their knowledge is tested in more challenging situations.

That it’s varied, it’s very instructive, it never gets monotonous, and it never gets old. There are always new things happening all the time. Both in the way we work and how we handle our duties. The fact that the tasks themselves are varied means that you never really stop learning (nurse, surgical outpatient care).

### Workplace and personal space balance

If nurses are to recover properly and cope with their work, they need to find a balance between work and leisure. This means having an active leisure time and feeling good at home while having the energy to deal with patients.

If you have a good home life and leisure time, it’s reflected in your work, then you can do a good job. If you’ve had a good weekend at home and feel that it’s given something, then you’re more rested and positive when you come to work (nurse, medical inpatient care).

The nurses need to be able to leave their duties behind and feel they are “finished” with them at the end of the workday. This entails being able to leave their work behind, both physically and mentally, so that it does not accompany them home and affect their leisure time.

Work must not take over so that I can’t cope during my spare time, and the same thing, leisure time should be energizing and help me cope even better at work, so that there’s a balance there. So that I feel I have time for both in a good way (nurse, psychiatric inpatient care).

The situation at home can affect nurses if they do not have enough energy for work. If there is an open, accepting atmosphere in the working team, the nurses can obtain support from colleagues and thus manage their work situation more easily.

You have to feel good about your social life at home. But it should be okay, if you have problems at home, that you don’t have to forget them when you come to work/…/What’s nice about my job is that I can come here and tell somebody that I've had a hard night or whatever it might be (nurse, surgical outpatient care).

The nurses report a need to have work schedules that enable a balance between work and leisure. When there are opportunities to have individual schedules, the chance of improving that balance is increased.

I’ve been working daytime for three years. We try to reschedule as best we can, so that I work maybe only ten evenings and as much daytime as possible. It works very well at home, when my husband works irregularly. Then, I’m at home if he works early mornings. And when my husband works late evenings, I’m the one who can pick up the children and manage the practical things (nurse, psychiatric inpatient care).

Having a schedule that suits me and that lets me recover. I have a little difficulty working evenings and getting up early. I’ve changed my schedule, so I work evenings before my day off (nurse, psychiatric inpatient care).

### Collaboration and supportive leadership

Interacting with others is an important precondition for managing the work situation. In interaction with others in the team, the nurses have the opportunity to obtain support, which helps them handle different care situations. Nurses can also receive support and expert help from other professionals, such as doctors, physiotherapists and auxiliaries.

*We work well together, we all work together, support each other and discuss things. We cooperate really well, I think. If there’s something you don’t know how to do, then you’re not afraid to get help from someone else, or to say "I can’t, but…" It challenges you, I think that’s really important (*nurse, psychiatric inpatient care*).*

For nurses to develop good manageability, clear leadership is needed and, thus, a clear and strong manager is required. Such leadership provides security and is a precondition for the work team to function satisfactorily.

A good manager makes the whole team work; she does almost everything for us, I almost said. She plays around with the schedule/…/she makes a special schedule for those who work until five and some work every other weekend, then they have special schedules. She really spends time and energy on us, which makes people want to stay and enjoy being on the ward, and you gain confidence (nurse, psychiatric inpatient care).

### Comprehensibility

Creating sustainable work situations for nurses means making these situations comprehensible. Comprehensibility is created when nurses can reflect on their role and the work they do. Comprehensibility helps nurses understand that they are needed and that they are providing good care for their patients. The nurses need varying tasks so that their work is not trivial and monotonous. Nurses develop and learn when they face new challenges on a daily basis. In this way, the professional role is constantly evolving. The nurses are proud of their professional role, and their professional role and function are valuable in the healthcare organization.

### Valued role and good work

The nurses understand that their professional role is of great value owing to the knowledge they have and the tasks they perform, which involve great responsibility. They feel needed and that they are contributing something important to patients’ health.

To me, my professional role means feeling I am capable. Having sufficient knowledge so that I can help people and have the energy to help when someone is powerless. Being able to support that person is a strong driving force for me (nurse, psychiatric inpatient care).

I’m thriving as a nurse, I enjoy meeting new people, and I enjoy helping, doing something for someone else, and as you can see... it usually turns out good for them (nurse, surgical outpatient care).

Sometimes, it is only the feeling of doing something that benefits someone else (the patient) that gives this satisfaction.

Yes, being able to help and provide support gives me satisfaction that it is working in all ways for our patients (nurse, psychiatric inpatient care).

It is the best profession in the world. Being able to help someone who needs it and to alleviate problems or support or treat in the best way. That's what I want to do (nurse, medical outpatient care).

### Commitment and involvement

The nurses feel highly committed to their work, which means they get deeply involved in their patients and try to do that “little extra” for them. It is important for nurses to not only carry out basic nursing, for example, patient hygiene care, but also help patients by talking with them and building trusting relationships.

I try to do that little extra for the patients, *"*to give of oneself.*"* If I get good treatment in return, it means a lot to me. I hear someone say, you did great, yes, it means a lot to me (nurse, medical outpatient care).

At the same time, the nurses understand that they cannot get too deeply involved in patients’ situations; they must also think about themselves. This is an approach they have developed over a long period of time to prevent work-related stress and ill health.

*You have to take care of yourself, not get involved in everything. It’s important that you don’t do everything yourself, that you can ask others for help and know your limitations, I think that’s important. That you don’t wear yourself out, because that helps nobody if you have to stay at home sick just because you’ve gotten too involved. Somehow, it’s about getting involved in the right things (nurse,* psychiatric inpatient care*).*

### Pride in professional role

The nurses are proud of their professional role and the responsibility it entails. According to the nurses, their expertise is also of great value, and this further strengthens their pride.

I’m proud of my professional role. I think like this, it’s a professional role, you get a salary and you get to work, earn money, then you can manage your life (nurse, medical inpatient care).

The nurses are proud of and have positive attitudes towards the work they do and its effects.

I’m proud of what I and my colleagues do. What I know we achieve and what we do for the patients. I’m incredibly proud of this (nurse, medical outpatient care).

## Discussion

The aim of the present study was to explore and describe factors explaining why hospital nurses remain in the workplace. In the study, nurses expressed a strong feeling of togetherness and pride in their profession, even under conditions involving stress and high workload. They highlight the importance of giving social support to and getting support from colleagues and patients, as this helps them manage daily life. They try to do the best they can for their patients, which gives them meaning in life and as professionals. The results reveal that nurses perceive an SOC and that this is important to their wellbeing.

Nurses’ engagement in patient care and work is consistent with previous findings showing that nurses thrive when they find meaning in their work [13]. Important to job engagement are feeling an appetite for work and vitality.

The nurses clearly describe a care-related driving force that is the source of their great commitment to their work. There are also clear altruistic elements involved, such as wanting to contribute something extra and not just performing tasks in a technical or routine manner. This influences their experience of doing something good and giving that ‘little extra,’ which is largely based on personal commitment to patients’ needs. The nurses experience a strong affinity with the working group and the collective, from which they receive a great deal of support and energy, which help them cope with everyday tasks.

Although the nurses in the present study report feeling highly committed to their work and patients, they must not get too involved. The nurses try to set limits – an approach they have developed over a long period to prevent work-related stress and ill health. In this way, they identify strategies for managing stress as well as for creating and maintaining an SOC [14]. Factors that can maintain nurses’ good health in stressful work environments are strengthening nurses’ professional pride, stressing the great value of their knowledge and duties, reinforcing their feeling of being needed and doing good, increasing opportunities for them to work with nursing duties such as talking with patients and building trusting care relationships, and creating an SOC that promotes meaningfulness, manageability and comprehensibility in work. Previous research has stressed the importance of job engagement, support from colleagues, personal characteristics and self-knowledge [13].

The results demonstrate that to achieve good recovery, nurses need to be able to leave work behind them, both physically and mentally, after their workday. The nurse manager can give nurses the opportunity to take some time for reflection before the end of the workday [23]. Hence, reflection can serve as a sustainability tool, helping nurses develop a “thought respite” and recovery, which can in turn help them control their work situation, provide extra energy to manage workloads and build a defence against stress – all of which can be sustainable in the long term.

According to the nurses in the present study, having a reduced workload, varied tasks, individual schedules, clear leadership and cooperation between nurses and other professionals are factors that contribute to a good working climate, SOC, and meaningfulness. These factors are important for nurse managers to consider in their efforts to support nurses’ meaningfulness in their work and make nurses’ work manageable. The present results are in line with findings from Furunes, Kaltveit and Akerjordet’s study [24], which showed how nurse managers can create a good working climate for nurses by giving them opportunities to participate in decision-making, develop their skills and provide social support; such a working climate can encourage nurses to remain in the job as valuable assets in the organization.

By raising awareness of the importance of the three SOC dimensions of meaningfulness, manageability and comprehensibility for nurses and nurse managers, we can acquire an understanding of and knowledge about these dimensions as well as develop skills that promote a better workplace and work environment [25]. In one study aimed at exploring how engaged people master intensive work through coping and job crafting in a salutogenic way, two strategies were identified – i.e., an active strategy and a cognitive strategy – involving both coping and job crafting. The specifics of these strategies determine whether salutogenic processes are present [26]. Elements of the active strategy were prioritizing work, working more hours, and seeking support from management or colleagues ([25] , p. 634), while attitudes, redefining thoughts about work, and hoping for a better future were elements of the cognitive strategy ([25] , p. 634).

Reflecting on the content of nursing practice and discussing with colleagues how to manage their work situation while providing good patient care involve experience-based learning processes. This approach is a form of work-integrated learning. In this way, the workplace and the nurse manager give nurses as individuals and as a professional group the opportunity to develop a learning process together with others [27], i.e., an SOC, which can also ensure that nurses develop experience-based knowledge generated through many years of working with patients and teams at the same workplace. This applies to their familiarity with technical skills and the caring approach. Experience-based knowledge is also an important basis for person-centred care and patient safety. The working group is an important resource for learning and sharing experiences; the nurse manager is important in that he/she can implement new approaches and strengthen the three SOC dimensions – meaningfulness, manageability and comprehensibility – thus allowing nurses to increase their job satisfaction and create a sustainable working life.

To create a good workplace for nurses, the nurse manager can develop action programmes for various activities, such as individual schedules, skill development, and cooperation between nurses and other professionals, to reduce the workload and reinforce clear leadership. The action programmes can promote a sustainable working life and prevent nurses from terminating their employment. In this way, nurses can develop experience-based knowledge generated through many years of working with patients and teams at the same workplace. This also applies to familiarity with technical skills and the caring approach. Moreover, experience-based knowledge is an important basis for person-centred care and patient safety.

### Strengths and limitations of the study

The most important strength of the present study is the focus on the nurse perspective. The application of the salutogenic theoretical model of health throughout the various stages in the study was also a strength [11]. To obtain a broad and comprehensive description of the study aim, that is, to describe and explore salutary factors promoting a sustainable working life for nurses, nurses from various care units were selected. The researchers’ backgrounds (five of the six researchers are nurses) may also be an advantage in understanding, analysing and interpreting the success factors promoting a sustainable working life for nurses. The authors are all familiar with and deeply grounded in the salutogenic theory of health, which facilitated both the inductive and deductive analysis process.

There is always a certain degree of interpretation when a researcher approaches a text, and therefore the text always has different meanings; this is an important issue regarding the trustworthiness of qualitative content analysis results [20, 21]. An important limitation is that all the participants were female nurses working in a hospital. No nurses from community care were included. As such, the finding might be limited to female nurses working in hospitals in Sweden. Another limitation is that no nurses who had already left the profession or who might have been thinking about leaving were included. The transferability of the present results to similar settings in other countries is limited because health care services are organized differently across settings and countries.

## Conclusion

The main findings of this study have shown the critical importance of being in a meaningful, comprehensible and manageable work context that supports nurses in maintaining their professional identity. This can be achieved by creating work environments where nurses feel pride in their professional roles in an organization that supports collaboration and togetherness with colleagues and leaders. An important prerequisite is to feel involved and have opportunities to influence their work situation.

## Data Availability

The datasets generated and analysed during the current study are not publicly available due to the participants’ requests for confidentiality and will not be shared. However, an aggregated copy of the data can be obtained from the corresponding author.

## Abbreviations

SOC: Sense of Coherence
COREQ: Consolidated Criteria for Reporting Qualitative Research
