# Correction to: Salutary factors and hospital work environments: a qualitative descriptive study of nurses in Sweden

**DOI:** 10.1186/s12912-021-00536-z

**Published:** 2021-01-21

**Authors:** Håkan Nunstedt, Monica Eriksson, Ayman Obeid, Lisbeth Hillström, Anh Truong, Sandra Pennbrant

**Affiliations:** Department of Health Sciences, University West, Trollhättan, Sweden

**Correction to: BMC Nurs 19, 125 (2020)**

**https://doi.org/10.1186/s12912-020-00521-y**

Following publication of the original article [1], the authors identified an error in the author names of Håkan Nunstedt, Monica Eriksson, Ayman Obeid, Lisbeth Hillström, Anh Truong and Sandra Pennbrant. The given name and family name were erroneously transposed.

The incorrect author name is: Nunstedt Håkan, Eriksson Monica, Obeid Ayman, Hillström Lisbeth, Truong Anh and Pennbrant Sandra.

The correct author name is: Håkan Nunstedt, Monica Eriksson, Ayman Obeid, Lisbeth Hillström, Anh Truong and Sandra Pennbrant.

The author group has been updated above and the original article [1] has been corrected.
